# Clinical Implementation of Automated Treatment Planning for Rectum Intensity-Modulated Radiotherapy Using Voxel-Based Dose Prediction and Post-Optimization Strategies

**DOI:** 10.3389/fonc.2021.697995

**Published:** 2021-06-24

**Authors:** Yang Zhong, Lei Yu, Jun Zhao, Yingtao Fang, Yanju Yang, Zhiqiang Wu, Jiazhou Wang, Weigang Hu

**Affiliations:** ^1^ Department of Radiation Oncology, Fudan University Shanghai Cancer Center, Shanghai, China; ^2^ Department of Oncology, Shanghai Medical College, Fudan University, Shanghai, China; ^3^ Shanghai Key Laboratory of Radiation Oncology, Shanghai, China

**Keywords:** automated treatment planning, dose distribution prediction, clinical post-optimization strategies, rectal cancer, intensity-modulated radiotherapy

## Abstract

**Purpose:**

This study aims to demonstrate the feasibility of clinical implementation of automated treatment planning (ATP) using voxel-based dose prediction and post-optimization strategies for rectal cancer on uRT (United Imaging Healthcare, Shanghai, China) treatment planning system.

**Methods:**

A total of 180 previously treated rectal cancer cases were enrolled in this study, including 160 cases for training, 10 for validation and 10 for testing. Using CT image data, planning target volumes (PTVs) and contour delineation of the organs at risk (OARs) as input and three-dimensional (3D) dose distribution as output, a 3D-Uet DL model was developed. Based on the voxel-wise prediction dose distribution, intensity-modulated radiation therapy (IMRT) plans were then generated automatedly using post-optimization strategies, including a complex clinical dose target metrics homogeneity index (HI) and conformation index (CI). To evaluate the performance of the proposed ATP approach, the dose-volume histogram (DVH) parameters of OARs and PTV and the 3D dose distributions of the plan were compared with those of manual plans.

**Results:**

By combining clinical post-optimization strategies, the automatically generated treatment plan can achieve better homogeneous PTV coverage and dose sparing for OARs except the mean dose for femoral-head compared with the use of the mean square error objective function alone. Compared with the manual plan, no statistically significant differences in HI, CI or global maximum dose were found. The manual plans perform slightly better than plans with post-optimization strategies in other dosimetric indexes, but these plans are still within clinical requirements.

**Conclusions:**

With the help of clinical post-optimization strategies, the proposed new ATP solution can generate IMRT plans that are within clinically acceptable levels and comparable to plans manually generated by dosimetrists.

## Introduction

Intensity-modulated radiotherapy (IMRT) is an important treatment modality and has been widely used for many types of cancer (1). This radiotherapy technology has the advantage of achieving higher dose coverage of planning target volumes (PTVs) with steep dose gradients at the transition to orangs at risk (OARs). The design of the IMRT plans requires several optimization cycles with manual adjustments of the weights and the addition of new optimization objectives to meet the clinically specific criteria. It is a time-consuming and labor-intensive process and extremely dependent on the experience of the designer. Consequently, the quality of a plan depends drastically on planners (experience, skill and available time) (2). This variability may lead to suboptimal plans, resulting in a negative impact on tumor control in patients (3, 4).

Recently, deep learning (DL) has been used to automate treatment planning and has received considerable attention in the radiotherapy community (5, 6). An approach to reducing user variability and improving the quality and efficiency of treatment plans is the use of so-called knowledge-based planning (KBP). KBP has been implemented in the commercial treatment planning system (TPS) Eclipse as the RapidPlan module (Varian Medical Systems, Palo Alto, CA). This strategy uses a large number of previous acceptable or superior clinical patient databases to estimate specific dose metrics or dose-volume histograms (DVHs) for a new patient (7–11). The main limitation of this method is the lack of spatial information, and planners still need to use hand-crafted features from statistical analysis (9, 12–19). To solve this problem, DL-based 3D dose distribution prediction techniques for automated treatment planning (ATP) have become a major focus of research. Based on popular convolutional neural networks (CNNs), the patient-specific three-dimensional (3D) dose distribution can be achieved and later used as an objective to generate a treatment plan automatically. Accurate spatial dose distribution prediction can eliminate dependence on handcrafted features completely and potentially improve plan quality and consistency (20–22).

However, the clinical implementation of current DL-based ATP solutions remains stymied by two points. First, the performance of the DL method strongly depends on the database used for training. The optimal result of the automated generated plan is to make each voxel dose the same as the predicted dose. Thus, the current voxel-based dose prediction methods cannot obtain a better plan, or the plan quality is limited by the predicted dose results. Second, given the high safety requirements of medical applications, generating executable clinical automated treatment plan based on 3D dose distribution in closed commercial software architecture remains a challenge. To the best of our knowledge, previous studies have generally focused on algorithms to improve the accuracy of 3D dose distribution prediction (21–23) or have used the open-source toolkit matRad to generate radiation treatment planning for educational purposes and research (20, 24).

Consequently, a DL model was developed for predicting a 3D dose distribution in this study. To decrease the quality limits of the training data, clinical post-optimization strategies were used in the process of automated plan generation based on the 3D prediction dose. We extensively collaborated with United Imaging Healthcare (UIH) Co., Ltd. (Shanghai, China) to achieve the clinical implementation of ATP in its TPS uRT.

## Materials and Methods

### Patient Data Collection

A total of 180 rectal cancer patients undergoing radiotherapy between 2017 and 2019 in our cancer center were enrolled in this study. For analysis, data were randomly divided into 160 training sets, 10 validation sets and 10 testing sets. Simulation CT images (slice thickness 5 mm; 512×512 matrix) were acquired using a Philips Brilliance Big Bore multidetector-row spiral CT scanner (Philips Healthcare, Cleveland, OH). Radiation oncologists delineated the gross tumor volume (GTV), clinical target volume (CTV) and OARs in the planning CT. The prescription of each patient was 50 Gy in 25 fractions (2 Gy per fraction). The IMRT treatment plans were calculated and optimized in Pinnacle 8.0-9.10 TPS (Philips Radiation Oncology Systems, Fitchburg, WI, USA). All these treatment plans were generated with 9 equiangular 6-MV photon beams.

### Model Architecture

**Figure 1:**
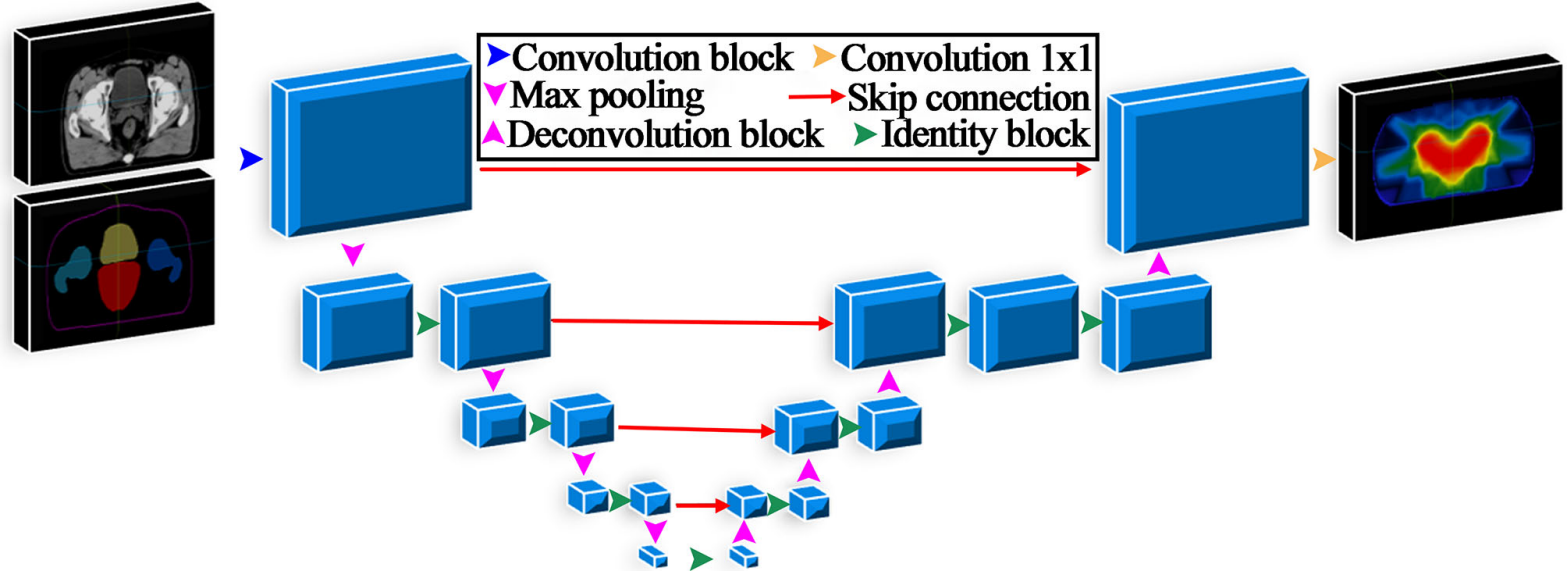
Architecture of the 3D U-Net used for dose prediction.

A 3D U-net was developed in this study and is illustrated in **Figure 1**. The number of filters for each convolutional layer was 32, 64, 128, 256, and 512, and the feature map size was reduced by half after the max-pooling layer. All convolutional layers applied a 3×3×3 kernel except the output layer with a 1×1×1 kernel. The input data were CT images and contours of regions of interest (ROIs), including the PTV, body bladder, left femoral head and right femoral head. The output data were the 3D predicted dose distribution. The model was implemented in Keras, and the loss function used in the training process was the mean square error. The Adam optimization algorithm was used to minimize the loss function value between the predicted dose and the clinical truth. The network parameters of the mode were initialized using the He_normal initialization method (25).

### Automated Planning

**Figure 2:**
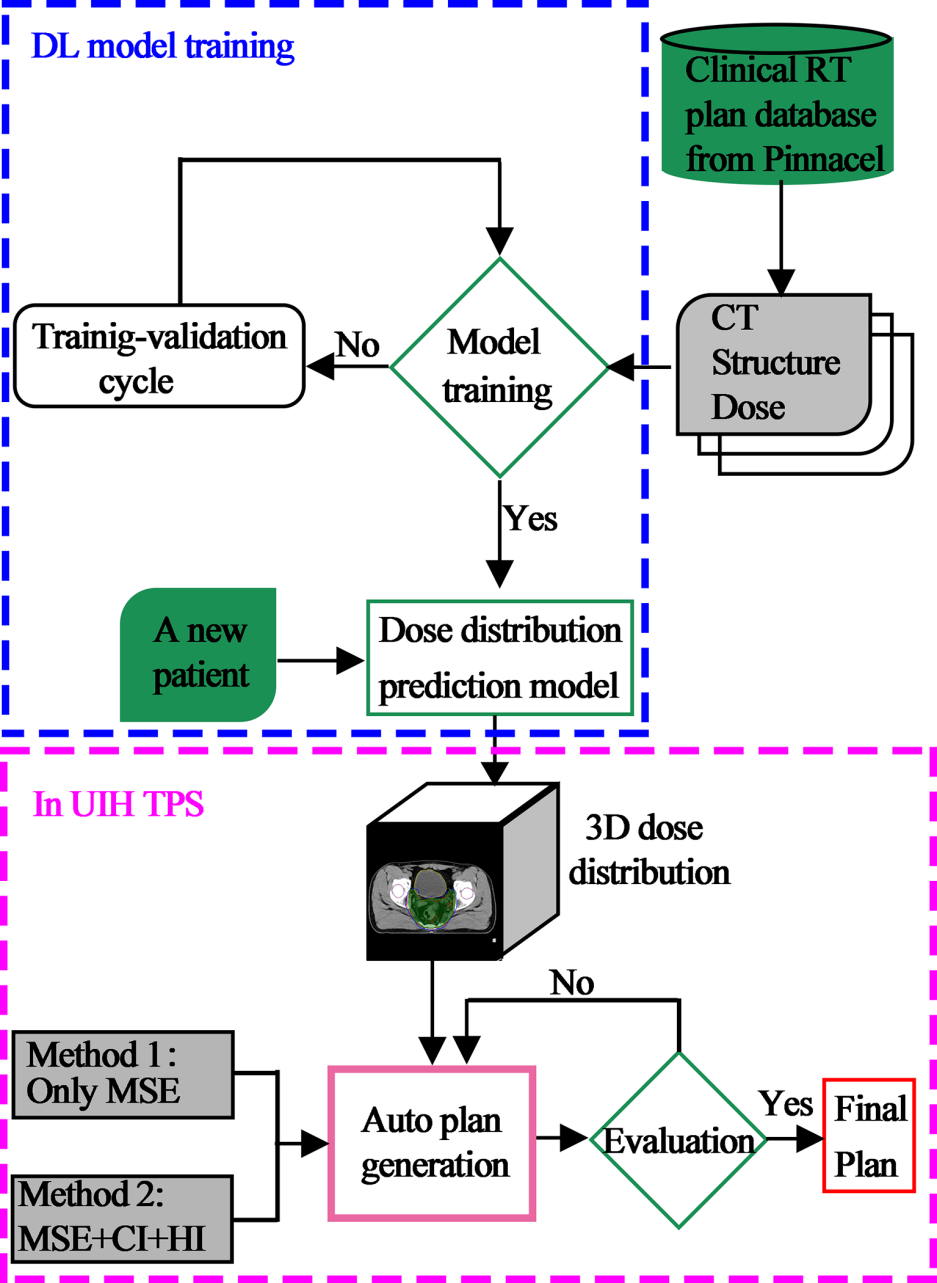
Overview of the automated treatment planning workflow.

To provide a more complete picture, the overall description of the framework of this paper is displayed in **Figure 2**. First, using previously treated plans generated and optimized in Pinnacle TPS as training data, a DL model was developed for predicting a 3D dose distribution. Second, a new patient CT with contouring information of the target and OARs was input to automatically generate the 3D dose distribution of the current case by our DL model. Third, based on these 3D dose predictions, automated IMRT plans were then generated using two methods in UIH TPS. One approach uses the mean square error (MSE) optimization function only. Another combines MSE and clinical post-optimization strategies, which include the complex clinical dose target metrics homogeneity index (HI) and conformation index (CI). All plans were generated automatically using 9 equally spaced fixed coplanar 6-MV photon beams in UIH TPS.

The function of MSE is described as follows:

 (1) $$F_{MSE}=\sum_{i\in V}{\left(d_{i}^{calc}-d_{i}^{pred}\right)}^{2}$$

where V denotes all voxels, *d_i_*denotes the dose of i^th^ voxel, and *d^calc^* and *d^pred^* represent the input and prediction dose in the iteration process, respectively.

The optimization function of HI is described as follows:

 (2) $$F_{obi}=W_{HI}*[{\left(D_{98}-D_{50}\right)}^{2}+{\left(D_{2}-D_{50}\right)}^{2}]$$

where *W_HI_* is the weight of the objective function, and *D_X_* is the corresponding dose of X% volume of the target in each iteration.

The optimization function of CI is described as follows:

 (3) $$F_{obi}=W_{CI}*{\left(f_{CI}-C_{index}\right)}^{2}$$

(4) $$F_{CI}=TV_{RI}*\frac{TV_{RI}}{\left(V_{RI}*TV\right)}$$

where *W_CI_* is the weight of the objective function, TV is the volume of the target, *V_RI_* is the volume of the reference isodose, T*V_RI_* is the interaction volume of the reference isodose and TV, and *C_index_* is constant (typically fixed as 1).

The total normalized objective function is noted as follow:

 (5) $$F_{total}=\alpha F_{MSE}+\sum_{i\in D}\beta_{D}F_{D}$$

where *F_total_* denotes the total objective function; α and β are the weight of the function of MSE and the clinical optimization index, respectively; and D represents the different index.

### Dosimetric Plan Evaluations

To evaluate the performance of the two proposed ATP methods, the DVH parameters of OARs and PTV and the 3D dose distributions of plan were compared between the ATP and original clinical plan. The clinically relevant dosimetric indexes, including the mean dose (Dmean), D2, D5, D95, and D98 for PTV (here, Di means the dose received by i% of PTV volume) and Dmean, V15, V25, V35, and V50 for OARs (here, Vi means volume fraction of OARs irradiated by i Gy), were calculated. HI (26) and CI (27) for PTV were further calculated using the following formulas:

 (6) $$\text{HI}=\frac{{\text{D}}_{5}}{{\text{D}}_{95}}$$

(7) $$\text{CI}=\frac{{\text{V}}_{\text{R}}*{\text{V}}_{\text{R}}}{{\text{V}}_{\text{Ptv}}*{\text{V}}_{\text{p}}}$$

where V_PTV_ and V_P_ are the volume of the PTV and the prescription dose region, respectively, and V_R_ is the irradiated PTV volume of the prescription dose. These results were compared using a paired-samples t-test for the two models with p < 0.05 considered statistically significant.

## Results

**Figure 3:**
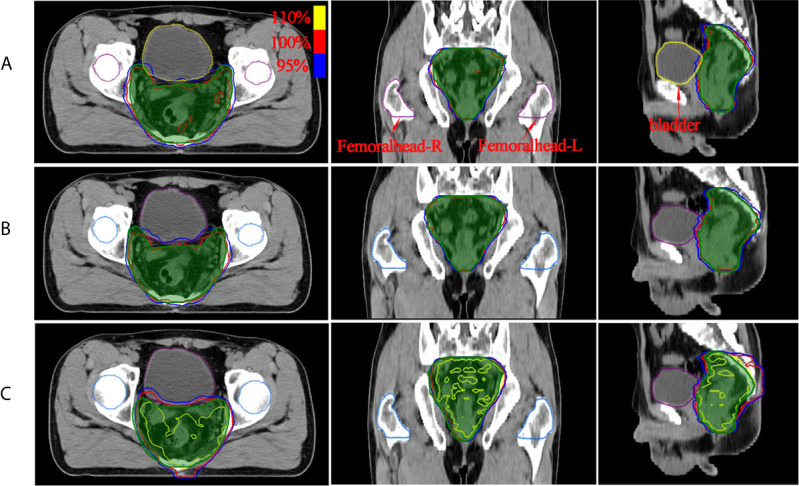
The dose distribution comparison of PTV region (green shaded area) for two methods with manual plan: **(A)** manual plan, **(B)** dose prediction based post-optimization plan and **(C)** MSE optimization plan. The unit of color bar is Gy.

**Figure 4:**
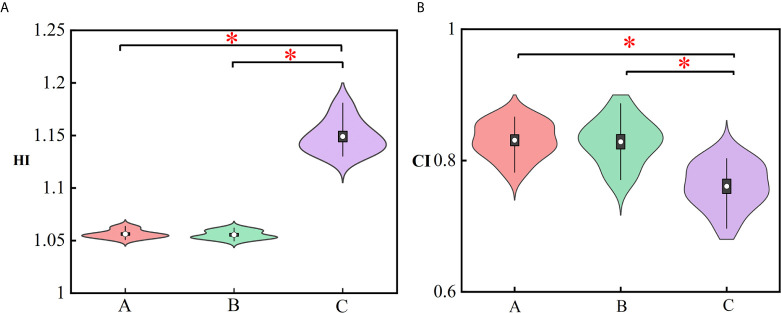
Violin plots representing the mean (white dot), σ (vertical rectangle), 95% percentile (black vertical line) and the probability distribution for the HI **(A)** and CI **(B)**. The asterisks represent p ≤ 0.05. (A) Manual plan, (B) dose prediction-based post-optimization plan and (C) MSE optimization plan.

**Figure 5:**
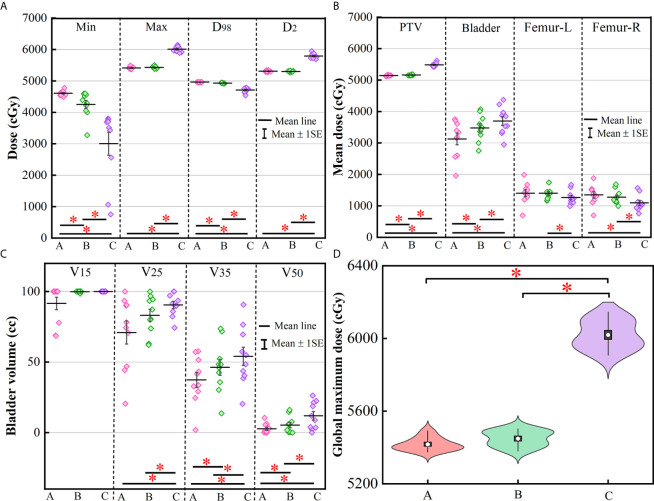
This graph displays a column scatter chart with the mean (±SE): **(A)** minimum, maximum, D_98_ and D_2_ of PTV; **(B)** mean value of PTV, bladder, femoral head-left and femoral head-right; **(C)** V_15_, V_25_, V_35_, and V_50_ for bladder; **(D)** violin plots of global maximum dose for three methods. The asterisks represent p ≤ 0.05. (A) Manual plan, (B) dose prediction-based post-optimization plan and (C) MSE optimization plan.

Isodose comparisons between the two plan-generated methods for a representative example case are shown in **Figure 3**. As evident in the graph, the MSE optimization plan (**Figure 3C**) delivered a substantially greater dose (110% of prescription with yellow line) to the PTV compared with the other methods. It is also worth noting that the plan (**Figure 3B**) using clinical post-optimization strategies resulted in approximately the same homogeneous PTV coverage as the manual plan (**Figure 3A**). To further illustrate the performance of the three methods. The OAR and PTV dose metrics are shown in **Figures 4**, **5** as violin plots.

**Figure 4** represents the violin plots for HI and CI. The performance of manual plans and dose prediction-based post-optimization plans were greater than those of MSE optimization plans. Significant differences were observed between MSE optimization plans and the other two approaches. For HI and CI, no significant differences were identified between manual plans and with clinical post-optimization strategies.

**Figure 6:**
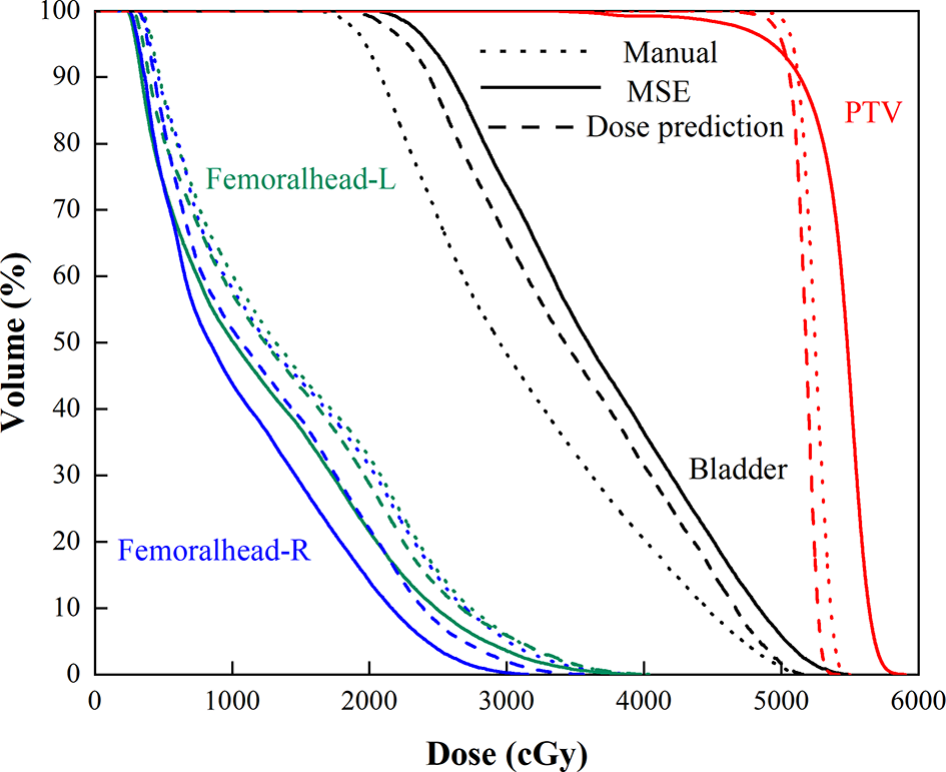
Mean DVHs of the manual plan (dotted line), MSE optimization plan (solid line), and dose prediction based post-optimization plan (dashed line).

More clinically relevant dosimetric indexes and DVH are shown in **Figures 5**, **6**, respectively. For dosimetric indexes of PTV, significant differences were observed between the MSE optimization plan and the other two methods. The MSE optimization plan did not outperform any dose metrics for PTV but had the lowest mean dose for femoral heads compared with the other two approaches. Of the specific dose metric constraints for the bladder, the manual plans achieved the lowest dose volume (V15, V25, V35 and V50) and mean dose followed by the dose prediction based post-optimization plans and then the MSE optimization plans. The global maximum dose doses of these four plans are presented in **Figure 5D**. Similarly, the MSE optimization approach was inferior to all methods and showed significant differences.

## Discussion

In this study, new optimization strategies directly related to clinical targets were used to improve the quality of ATPs. To our knowledge, this is the first study that implements clinical post-optimization strategies in the process of automated treatment planning for rectal cancer. We designed a deep learning model with clinical post-optimization strategies, as summarized in **Figure 2**. With the help of these intelligent strategies, the proposed optimization methods can pull high target dose and spare more OAR dose to obtain a better auto plan. This method can improve the plan quality of automated plans, which are limited by the quality of the training dataset.

There have been some studies on ATP techniques using DL neural networks (20–23, 28–30). Unlike conventional inverse optimization radiotherapy treatment planning with trial and error, ATP can be summarized into two steps: obtaining the predicted dose distribution and generating an automated executable plan (6). It was not easy to predict 3D voxel-wise dose distributions for IMRT plans in previous years due to the complicated relationship between OARs and PTV and the significant variability of PTV shapes. With the rapid advancement of machine learning, the accuracy of 3D dose distribution prediction methods has increased substantially. Compared with the DVH-based prediction algorithms, the prediction models have significant advantage in a way that it could provide spatial dose distribution information. These methods evolve from 2D model (28)to the 3D model (20, 29, 31, 32) and can provide a more robust predicted dose distribution.

However, these existing ATP strategies use the predicted 3D dose distribution as the end without any post-optimization. The optimal result of the automated generated plan is to make each voxel-wise dose the same as the predicted dose. Thus, current voxel-based methods cannot obtain a better plan due to two possible reasons. First, the performance of the deep learning-based model requires a sufficiently large, high-quality database. If the ground truth doses are suboptimal, the predicted doses will also be suboptimal as noted in the “garbage in, garbage out” paradigm. Second, more clinical conditions, such as significant variability in PTV size and complicated spatially neighboring anatomy, also have an impact on dose prediction. Previous studies require only the predicted dose distribution for optimization and did not take into consideration the diverse clinical scenarios for each patient. Therefore, it is challenging to achieve a specific plan for different patients, and the final plan had a worse homogeneity index of the target. Based on the above analysis, the proposed automated treatment planning strategy in our study serves as a step forward and provides a new idea to improve the performance of ATP.

The dose distribution in **Figure 3** shows that the method using clinical optimization strategies exhibited more accurate homogeneous PTV coverage than the MSE optimization plan. This conclusion is also strongly supported by more clinical interested dosimetric indexes found on **Figures 4**–**6**. These approaches can generate treatment plans that are within clinically acceptable levels and noninferior to plans manually generated by dosimetrists. The quality and consistency of treatment planning for radiotherapy can be largely improved.

However, there are some limitations to address in this study. We investigated the applicability of the clinical optimization of ATP for rectal cancer. The feasibility of the automated plan strategy should be demonstrated for nasopharyngeal cancer patients who have a rather complicated relationship between OARs and PTV and multiple targets, thus requiring different dose prescriptions. These are important priorities of future research.

## Conclusion

This paper demonstrates the feasibility of a new automated treatment planning strategy that includes clinical dose metrics post-optimization based on 3D dose distribution prediction. The approach can generate treatment plans that are within clinically acceptable levels and comparable to plans manually generated by dosimetrists. This methodology has great potential to improve the consistency and quality of IMRT planning by minimizing human intervention in the process of plan design.

## Data Availability Statement

The raw data supporting the conclusions of this article will be made available by the authors, without undue reservation.

## Author Contributions

Conception, design, and drafting the manuscript were performed by YZ, LY, JW, and WH. Data collection and interpreting were performed by YF, YY, and ZW. All authors contributed to the article and approved the submitted version.

## Funding

This work is supported the Shanghai Committee of Science and Technology Fund (19DZ1930902) and Xuhui District Artificial Intelligence Medical Hospital Cooperation Project (2020-009).

## Conflict of Interest

The authors declare that the research was conducted in the absence of any commercial or financial relationships that could be construed as a potential conflict of interest.
